# Delayed Posterior Fossa Hemorrhage Following a Tangential Gunshot Wound to the Occiput in a Patient With Chronic Liver Disease: A Case Report

**DOI:** 10.7759/cureus.70866

**Published:** 2024-10-04

**Authors:** Matthew D Tran, Jordan Davies, Alexander S Himstead, Gianna Fote, Joseph Rinehart

**Affiliations:** 1 Department of Anesthesiology and Perioperative Care, University of California Irvine Health, Orange, USA; 2 Department of Neurological Surgery, University of California Irvine Health, Orange, USA

**Keywords:** chronic liver disease, coagulopathy, gunshot head wound, head and neck trauma, neurogenic pulmonary edema, neurogenic shock, neurotrauma, posterior fossa hemorrhage, suboccipital craniectomy

## Abstract

Gunshot wounds (GSWs) to the head and neck are a common etiology of traumatic brain injury. Tangential GSWs (TGSWs) are a subset of GSWs wherein the missile penetrates tissue adjacent to the cranium, causing varying degrees of intracranial injury. Most patients sustaining TGSWs present with relatively benign neurological findings, and while a significant proportion have varying degrees of intracranial hemorrhage (ICH) on computed tomography, these tend to respond well to nonoperative management. We present a case report of a 28-year-old male who sustained a TGSW to the occiput, with a nonfocal neurological examination, small-volume posterior fossa ICH, a blunt vertebral artery injury (BVAI), and hepatic coagulopathy, who rapidly decompensated six hours after presenting due to massive posterior fossa hemorrhage with brainstem compression, requiring emergent cardiopulmonary resuscitation followed by suboccipital decompression and cerebrospinal fluid diversion. We propose that the patient’s BVAI led to an unexpected thromboembolic event, precipitating an ischemic stroke that underwent hemorrhagic conversion in the setting of coagulopathy. This case report emphasizes the insidious danger that TGSWs to the head and neck present to patients, and risk factors for poor outcomes, such as BVAI and coagulopathy. This report also highlights potential intraoperative challenges during surgery for acute mass lesions in the posterior fossa, such as neurogenic shock and pulmonary edema, that warrant careful consideration and preparation in neurosurgical cases.

## Introduction

Civilian gunshot wounds (GSWs) to the head and neck, resulting in traumatic brain injury, are common in urban settings in the United States and carry a high risk of morbidity and mortality [1,2]. Tangential GSWs (TGSWs) represent a subset of GSWs and have been described in the literature as an injury caused by a missile that passes through tissue adjacent to the cranium, causing intracranial injury without penetrating the cranium itself [3,4]. Case series of patients with TGSWs to the head have found that most present to the hospital without significant neurological deficits or altered sensorium. Computed tomography (CT) scans revealed intracranial hemorrhage (ICH) in a significant proportion of these patients, with intraparenchymal hematomas or contusions, subarachnoid hemorrhage (SAH), and subdural hematoma (SDH) as the most common subtypes [5,6]. At least one case of an SDH in the posterior fossa, secondary to a TGSW to the occiput, has been previously reported in the literature [7]. However, to our knowledge, no cases of delayed posterior fossa intraparenchymal hemorrhage following a TGSW to the occiput, in the context of underlying coagulopathy, have been reported.

Here, we present a case of a patient with chronic liver dysfunction who developed a devastating, delayed posterior fossa hemorrhage after suffering a TGSW to the occiput, requiring emergent suboccipital decompression and external ventricular drain (EVD) placement. This report will discuss possible mechanisms for this patient’s condition and the unique challenges encountered during management.

## Case presentation

A 28-year-old Hispanic male with no known past medical history was transferred to our Emergency Department as a critical trauma activation after sustaining a TGSW to the occiput. On arrival, he was hypertensive, with a blood pressure of 171/96 mmHg, but his vital signs were otherwise unremarkable. He was awake, alert, and oriented, without cranial nerve deficits, and had full power and sensation on examination. There was a 2 cm laceration to the right occipital area with a substantial underlying cephalohematoma, but no active hemorrhage. There were also small scattered metallic fragments on the superficial skin of the upper back. Laboratory values showed evidence of alcoholic hepatocellular injury (elevated aspartate aminotransferase (AST), alanine aminotransferase (ALT), and AST/ALT ratio) and impaired hepatic synthetic function (elevated total bilirubin and international normalized ratio (INR)). Other pertinent laboratory abnormalities included hyperglycemia (310 mg/dL), elevated HbA1c (8.1%), and elevated serum ethanol level (208 mg/dL) (Table 1).

**Table 1:** Pertinent lab results prior to decompensation ALP: alkaline phosphatase; ALT: alanine transaminase; AST: aspartate transaminase; ADP: adenosine diphosphate; PT: prothrombin time; INR: international normalized ratio; PTT: partial thromboplastin time; HbA1c: glycated hemoglobin

Lab	Patient’s value	Reference value
Metabolic panel		
Glucose	310	70-115 mg/dL
ALP	230	34-104 U/L
ALT	66	7-52 U/L
AST	161	13-39 U/L
Total bilirubin	3.0	0-1.4 mg/dL
Coagulation		
PT	16.6	9.4-12.5 s
INR	1.50	0.82-1.12
PTT	38.7	25.1-36.5 s
ADP/collagen	206	<112 s
Epinephrine/collagen	237	<169 s
Toxicology		
Alcohol	208	None
Other		
HbA1c	8.1	4.6-5.6%

**Figure 1:**
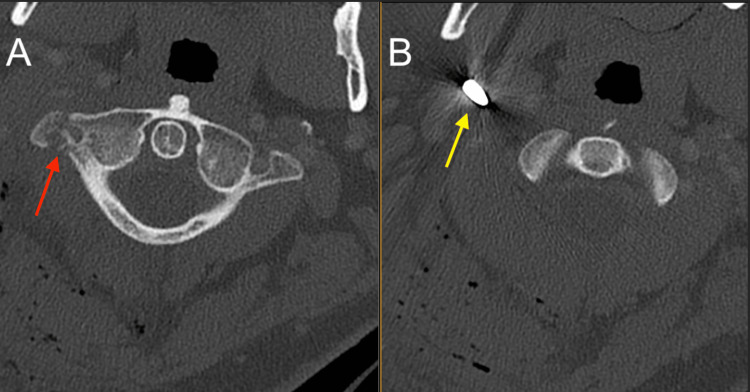
A CT scan of the cervical spine. (A) C1 right transverse process fracture (red); (B) Metallic foreign body (yellow) CT: computed tomography

**Figure 2:**
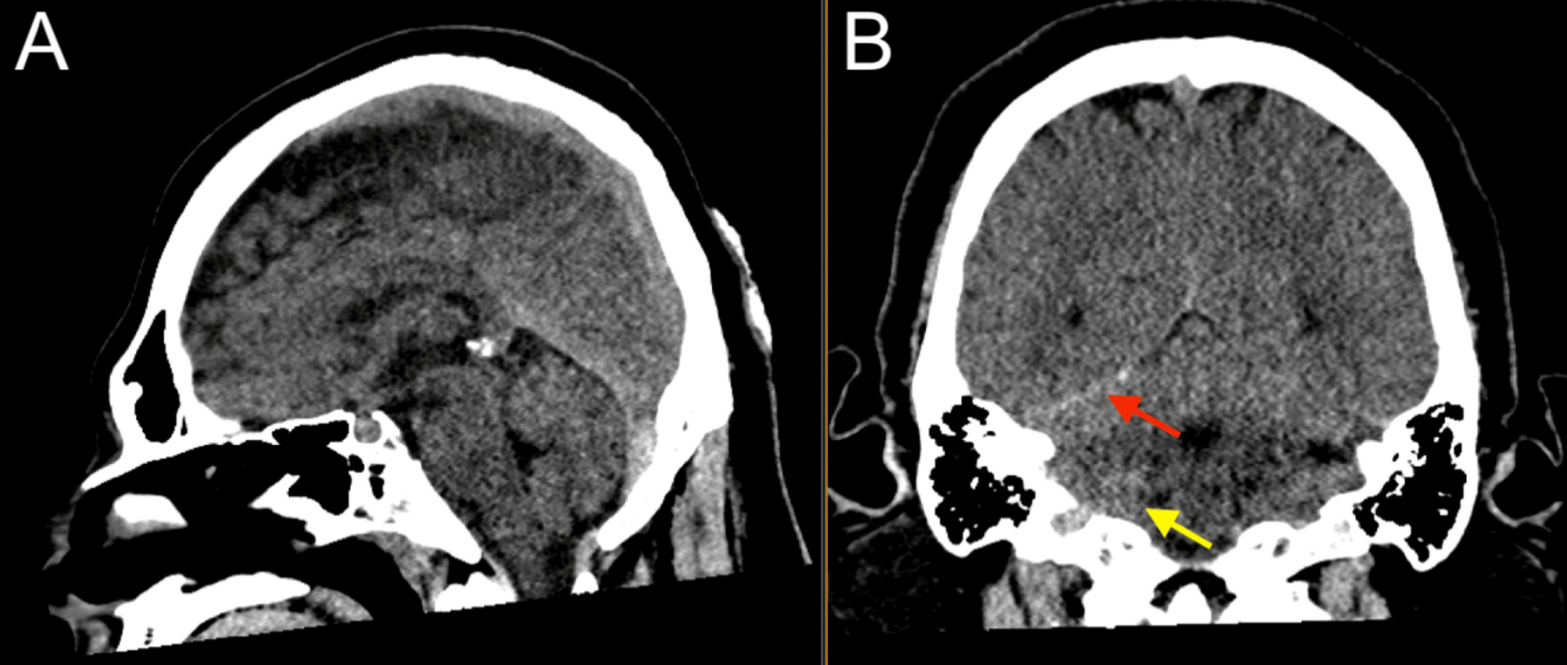
Head CT showing trace right posterior fossa and tentorial leaflet subdural hemorrhage (red), and trace right cerebellar subarachnoid hemorrhage (yellow). (A) Sagittal view; (B) Coronal view CT: computed tomography

**Figure 3:**
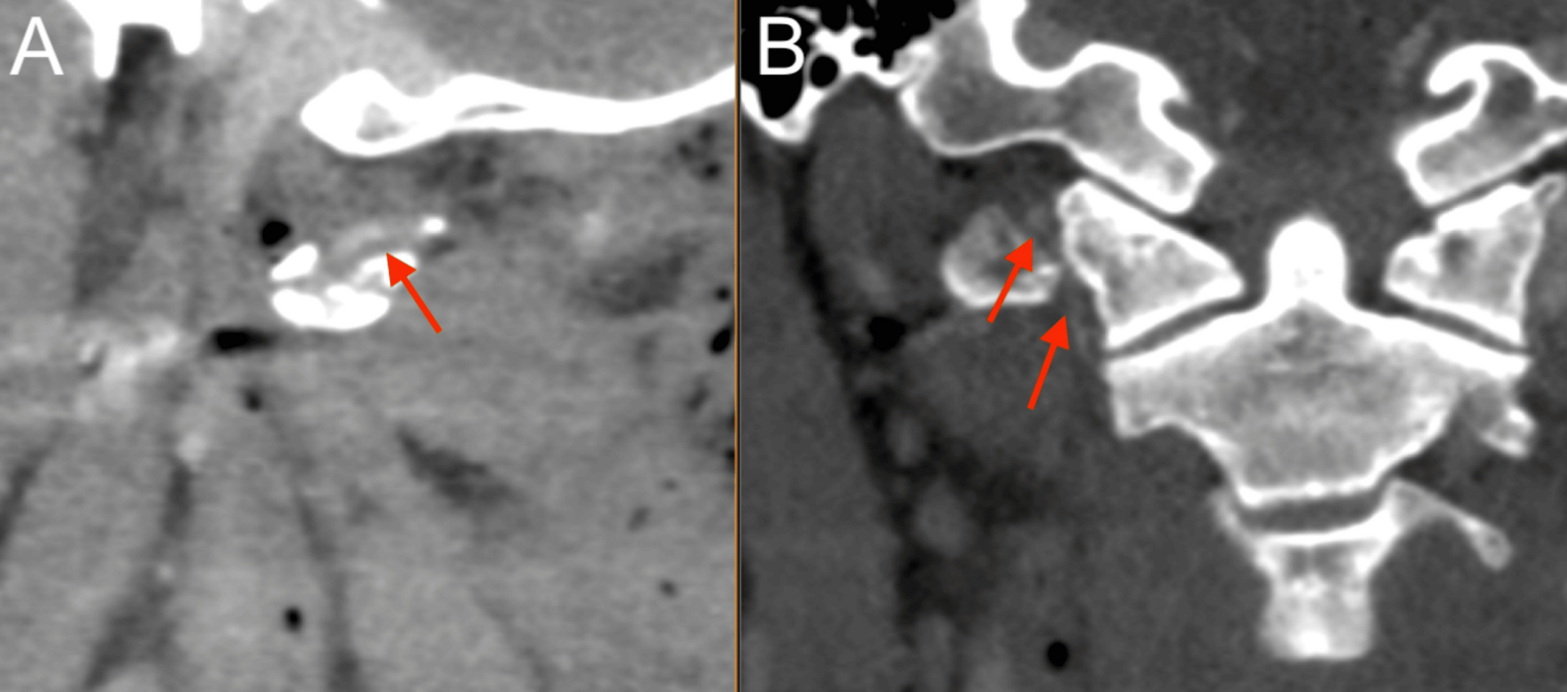
Neck CTA showing Biffl Grade 2 injury to right vertebral artery at level of C1-C2 (red). (A) Sagittal view; (B) Coronal view CTA: computed tomography angiogram

After the primary trauma survey, a noncontrast CT of the head and cervical spine revealed a prominent metallic foreign body lateral to the right carotid space at the level of the first cervical vertebra, trace hemorrhage in the right posterior fossa layering along the tentorium, and trace SAH (Figures 1-2). A CT angiogram (CTA) of the neck showed a nondisplaced fracture of the right C1 transverse process involving the transverse foramen (Figure 1), with a suspected Biffl Grade 2 injury of the right vertebral artery at the level of C1-C2 (Figure 3).

**Figure 4:**
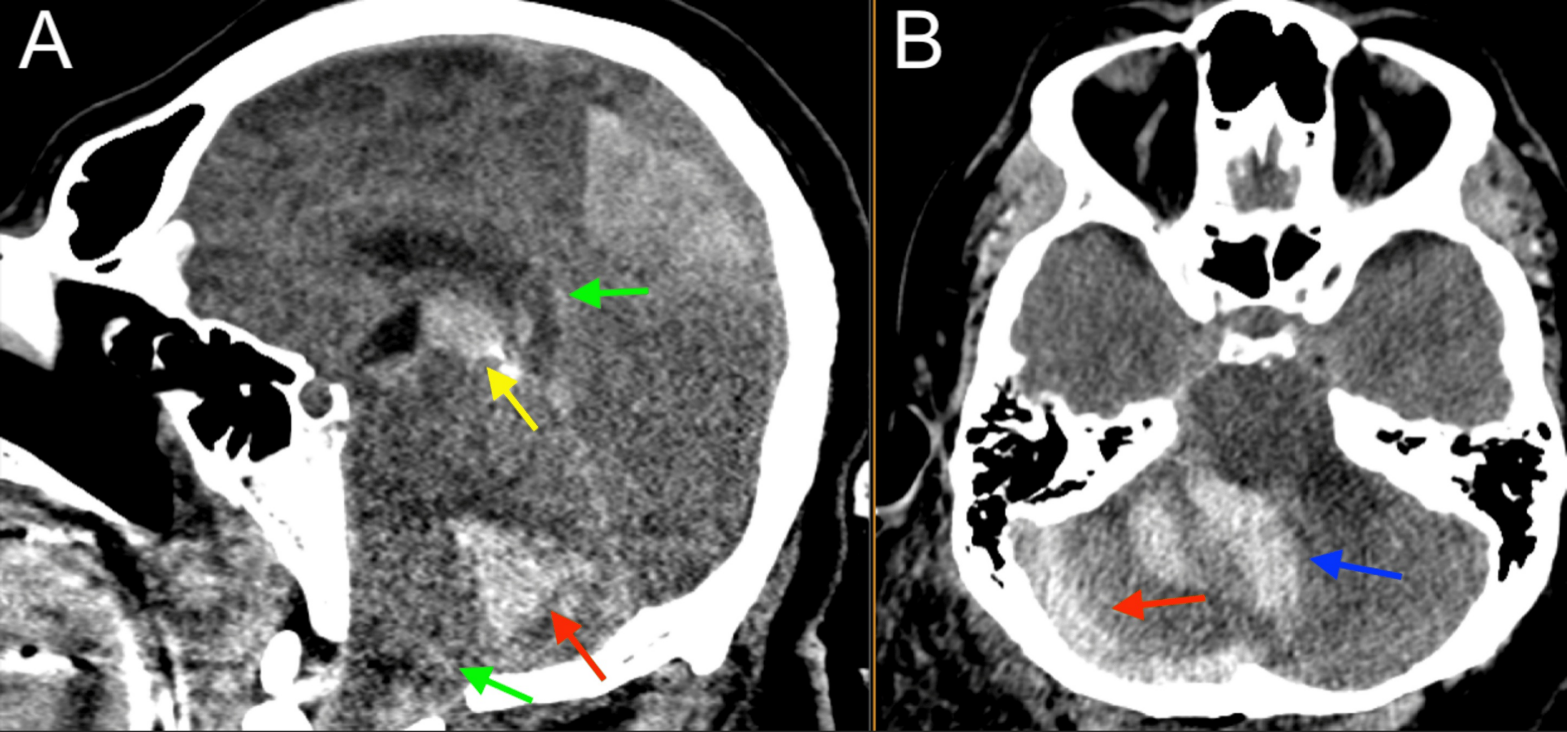
Head CT showing right cerebellar parenchymal hemorrhage (red), third ventricle intraventricular hemorrhage (yellow), fourth ventricle intraventricular hemorrhage (blue), foramen magnum crowding, and third ventricle upward herniation demonstrating posterior fossa fullness (green). (A) Sagittal view; (B) Axial view CT: computed tomography

**Figure 5:**
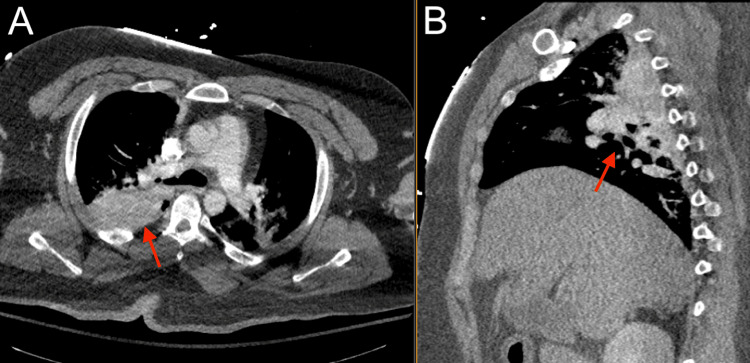
Chest CTA showing possible pulmonary hemorrhage, contusion, and atelectasis (red). (A) Axial view; (B) Sagittal view CTA: computed tomography angiogram

**Figure 6:**
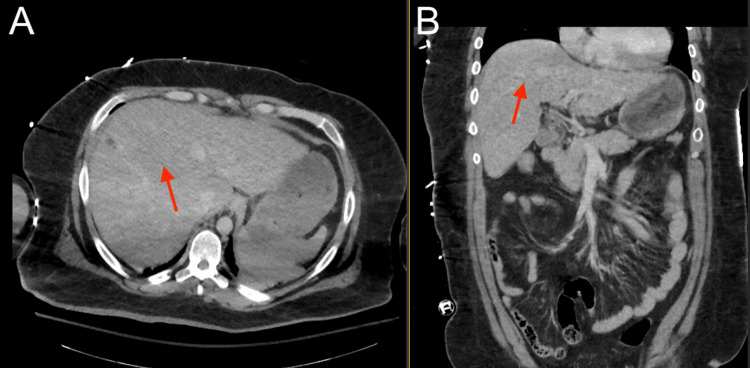
Abdominal CT showing suspected chronic liver disease (red). (A) Axial view; (B) Coronal view CT: computed tomography

Given the patient’s benign neurological examination and intracranial findings, a plan was made for nonoperative management with serial neurological checks and repeat imaging, and the patient was admitted to the surgical step-down unit. Low-dose aspirin (81 mg) was recommended for the Biffl Grade 2 vertebral artery injury but could not be started safely, given the high risk of hemorrhage. Platelet function analysis (PFA) was performed and was abnormal at this time (Table 1). The patient did not receive coagulopathy reversal with fresh frozen plasma or platelets. Six hours after the initial presentation, the patient suddenly developed agonal respirations and decompensated into pulseless electrical activity (PEA), prompting a code blue and activation of the advanced cardiac life support (ACLS) protocol. Return of spontaneous circulation (ROSC) was achieved after one round of cardiopulmonary resuscitation (CPR) with no medications, and the patient was intubated for airway protection. Stat neuroimaging obtained after intubation showed the interval development of a large right cerebellar intraparenchymal hemorrhage, increased fullness of the posterior fossa with brainstem compression and early upward herniation, and intraventricular hemorrhage of the third and fourth ventricles (Figure 4). Chest CTA revealed possible pulmonary hemorrhage, contusion, and atelectasis (Figure 5), and an abdominal CT revealed suspected chronic liver disease (Figure 6). The patient was taken immediately to the operating room for suboccipital decompression.

After a midline incision was made for generous exposure of the occipital bone and craniocervical junction, two burr holes just inferior to the transverse sinuses were created. The craniotome was used to turn a craniotomy flap measuring 3 cm in width on either side of the midline and extending from the transverse sinus down to the foramen magnum. The dura was opened sharply in a Y-shape, and the intraparenchymal hematoma was evacuated using suction and blunt dissection. Despite these measures, the cerebellum continued to swell out of the craniectomy defect, so an ultrasound-guided occipital EVD was placed at Frazier’s point, which had been previously prepped and draped into the field. 

During the operation, the patient went into shock, with his mean arterial pressure (MAP) falling below 50 mmHg, and required norepinephrine administration to maintain a MAP above 65 mmHg. Due to developing patient instability, the dura was left open, and a dural substitute was laid over the cerebellar hemispheres; the muscle and skin were closed expeditiously. At one point, we noticed copious bloody and frothy secretions in the patient’s endotracheal tube (ETT), affecting ventilation and suggesting acute pulmonary edema. This was resolved by suctioning the ETT, with immediate improvements in peak pressures and tidal volume. The surgery was otherwise successful, without any further complications, and the patient was kept intubated and taken to the Intensive Care Unit (ICU) in critical condition.

Post-operatively, the patient was in the ICU, requiring pressors for hypotension and experiencing another code event. The patient was resuscitated again and eventually stabilized. His neurologic recovery plateaued, reflecting severe brainstem injury, where the patient demonstrated intermittent wakefulness and followed simple commands with his eyes but was unable to move or respond to stimuli in his extremities. Communication in this manner was unreliable, and it was deemed that he was unable to understand the complex medical care being provided for him and his guarded neurologic prognosis. The family was engaged in goals of care discussions and indicated that he would want to have an independent and active lifestyle. To help guide prognosis, bedside somatosensory evoked potentials (SSEPs) were obtained, demonstrating an absent cortical response and indicating severe, likely irreversible brainstem injury, and a very low chance of motor or sensory recovery. These results helped guide the patient’s family in understanding long-term prognosis and decisions regarding goals of care. Other problems managed during the patient’s course in the ICU included acute hypoxic and hypercapnic respiratory failure, neurogenic and septic shock, ventilator-associated pneumonia, and bacteremia. Ultimately, after three weeks in the ICU, the patient’s family decided to proceed with comfort care. The patient was started on comfort-guided measures, compassionately extubated, and expired.

## Discussion

ICH is a common head CT finding in TGSW patients, even though most present with a Glasgow Coma Scale (GCS) of 15, no loss of consciousness, and a normal neurologic examination [5,6]. We present a case of a patient with chronic liver disease who suffered a TGSW to the occiput, who, despite having a relatively normal initial presentation and trace ICH findings on CT, rapidly developed a devastating posterior fossa hemorrhage requiring emergent suboccipital craniectomy and EVD placement. Worsening findings on repeat scans of patients with some initial intracranial pathology found on head CT have been reported, although they are uncommon [5,8]. However, our patient’s delayed acute decompensation in the context of underlying liver dysfunction warrants further discussion.

The devastating nature of this patient’s ICH, particularly from a missile that did not penetrate the cranium, was curious. The delayed onset of the ICH prompted additional questions about the progression of our patient’s condition. We postulate that the patient suffered an ischemic stroke in the posterior fossa caused by a thromboembolus originating from his dissected right vertebral artery. A systematic review of patients with vertebral artery dissection (VAD) by Gottesman et al. found that 68% of patients with extracranial (segments V1-V3) VAD developed ischemic stroke [9]. Biffl et al. reported a 24% rate of posterior fossa stroke in patients with blunt vertebral artery injury (BVAI), independent of Biffl injury grade. They speculated that patients with lower Biffl grades (nonocclusive) BVAI were more susceptible to ischemic stroke because the nonocclusive intimal injury may promote platelet-aggregation-mediated thromboembolus and subsequent infarction [10]. In the context of TGSWs to the head and neck, blunt arterial injuries should be considered when assessing stroke risk, and patients should be closely monitored and managed for possible thromboembolic events.

We suspect that the patient’s ischemic stroke subsequently underwent hemorrhagic conversion. Hemorrhagic conversion is a phenomenon that occurs in 18-42% of acute ischemic strokes and is thought to be due, in large part, to endothelial dysfunction and damage to the integrity of the blood-brain barrier (BBB), which increases vascular permeability. Hypertension, hyperglycemia, and thrombocytopenia are also associated with an increased risk of hemorrhagic conversion [11]. The patient’s lab data are consistent with these three risk factors, further supporting the plausibility of hemorrhagic conversion as the mechanism behind his acute decompensation and radiographic findings.

Sustaining a TGSW suggests that trauma-induced coagulopathy may have played a role in the severity of the patient’s posterior fossa hemorrhage [12]. However, more interesting is the potential role of his underlying chronic liver disease, which is not always seen in the context of trauma. Initial liver function tests performed on admission showed elevated liver enzymes and total bilirubin, raising suspicions of liver disease. These suspicions were confirmed by abdominal CT, revealing chronic liver disease. The etiology of this patient’s liver disease was likely due to chronic alcohol consumption, as he endorsed drinking six standard drinks per week, had an elevated AST/ALT ratio, and had no history of viral hepatitis. This diagnosis of chronic liver disease, in combination with the patient’s elevated coagulation tests and thrombocytopenia, suggested that he had some underlying cirrhotic coagulopathy. In coagulopathy associated with chronic liver disease, poor liver synthetic capacity causes a concomitant decrease in both circulating pro- and anti-coagulant factors. This produces a paradoxical hypo- and hyper-coagulable state with an unstable balance of coagulation and fibrinolysis, and the propensity for either depends on other underlying pathologies and the current clinical condition [13,14]. In the present case, the cirrhotic coagulopathy may have increased his risk for hemorrhagic conversion and the subsequent severity of his posterior fossa hemorrhage. Thorough investigation and identification of any pre-existing coagulopathies in patients experiencing or at risk for ischemic stroke or ICH may better inform their plan of care. 

During surgery, we also encountered physiological challenges requiring anesthesiologist intervention. First, the patient went into neurogenic shock and required administration of norepinephrine to maintain a MAP above 65 mmHg. This was not surprising, as hemodynamic instability, such as neurogenic shock, is a common consequence of upper spinal cord or brainstem injury due to the disruption of descending pathways between the central autonomic centers in the medulla oblongata and the sympathetic neurons in the intermediolateral thoracic and lumbar spinal cord [15,16]. In the setting of neurogenic shock, it is recommended that hypotension be treated by restoring intravascular volume, followed by administering vasopressors such as norepinephrine [15]. Second, the patient developed secretions due to what we presumed to be neurogenic pulmonary edema (NPE) that required suctioning of the ETT. The overall reported prevalence of NPE is relatively low, at 2-8%, though recent studies in patients with SAH suggest it may occur more frequently. Although pulmonary edema is generally quite common after neurologic disease, it is difficult to distinguish NPE from other forms. There are several suggested mechanisms behind the pathophysiology of NPE, such as neurally induced pulmonary vasoconstriction, transient left heart failure, and increased pulmonary capillary permeability under the influence of elevated intracranial pressure [17]. These intraoperative challenges demonstrate the need for anesthesiologists to anticipate and properly manage the non-neurological sequelae of ICH.

## Conclusions

In conclusion, patients with TGSWs to the head and neck are at risk for poor outcomes, even if their initial presentation and workup are relatively normal. Here, we propose a mechanism behind a devastating posterior fossa hemorrhage involving vertebral artery injury and subsequent hemorrhagic conversion of an ischemic stroke. Special attention should be paid during workup to blunt arterial injuries and markers for potential underlying coagulopathy, which may be risk factors for developing ischemic stroke and ICH. Anesthesiologists should also be aware of potential intraoperative challenges arising from ICH, such as neurogenic shock and pulmonary edema, and be prepared to rapidly intervene.
